# An Independent UAV-Based Mobile Base Station

**DOI:** 10.3390/s25051349

**Published:** 2025-02-22

**Authors:** Sung-Chan Choi, Sung-Yeon Kim

**Affiliations:** 1 School of Electrical and Electronic Engineering, Yonsei University, Seoul 03722, Republic of Korea; 2 Autonomous IoT Research Center, Korea Electronics Technology Institute, Seongnam 13509, Republic of Korea; csc@keti.re.kr; 3 Department of IT Convergence, ICT Polytech Institute of Korea, Kwangju 12777, Republic of Korea

**Keywords:** UAV, mobile base station, PS-LTE, IOPS, implementation, prototype

## Abstract

In disaster scenarios, e.g., earthquakes, tsunamis, and wildfires, communication infrastructure often becomes severely damaged. To rapidly restore damaged communication systems, we propose a UAV-based mobile base station equipped with Public Safety LTE (PS-LTE) technology to provide standalone communication capabilities. The proposed system includes PS-LTE functionalities, mission-critical push-to-talk, proximity-based services, and isolated E-UTRAN operation to ensure the reliable and secure communication for emergency services. We provide a simulation result to achieve the radio coverage of mobile base station. By using this radio coverage, we find an appropriate location of the end device for performing the outdoor experiments. We develop a prototype of a proposed mobile base station and test its operation in an outdoor environment. The experimental results provide a sufficient data rate to make an independent mobile base station to restore communication infrastructure in areas that experienced environmental disasters. This prototype and experimental results offer a significant step forward in creating agile and efficient communication solutions for emergency scenarios.

## 1. Introduction

In various disaster scenarios, e.g., earthquakes, tsunamis, and wildfires, existing communication infrastructure may be severely damaged. As a result, it would be impossible to monitor the disaster situation in real time, and we cannot communicate with personnel and equipment which are essential to recover the damaged area. This lack of communication capability makes the coordination and effectiveness of disaster recovery operations more difficult.

To resolve this lack of communication problem, in disaster scenarios, various approaches have been studied to establish communication networks that enable rapid response, cost-effectiveness and seamless connectivity in [1,2,3,4,5,6,7,8,9,10,11,12,13,14]. In [1,2,3,4], the papers focus on improving communication frameworks to reduce risks, mitigate psychological impacts, and engage vulnerable populations. Furthermore, the role of clear and accessible information is emphasized in enhancing disaster preparedness and responses. To maintain reliable communication in disaster scenarios where traditional infrastructure may be damaged or unavailable, in [5,6,7,8], novel communication protocols are introduced which can rapidly restore connectivity, support high-demand conditions, and operate under challenging environmental constraints. In [9,10,11,12], a drone is used as a communication platform in disaster scenarios. These studies aim to exploit the mobility and flexibility of drones to provide dynamic and adaptive communication solutions in areas where traditional infrastructure is unavailable or destroyed. In [13,14], the practical implementations of disaster communication systems are studied to bridge the gap between theoretical models and the implementations. These studies validate the proposed communication solutions through simulations or field experiments. The above studies have explored various aspects of communication recovery in disaster scenarios. However, they have not validated whether mobile communication can be reliably restored in real-world disaster scenarios.

**Figure 1:**
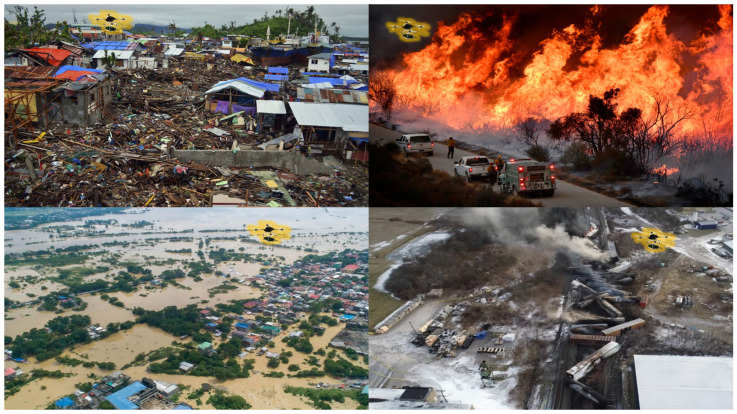
An example of UAV to restore the communication infrastructure in disaster areas.

Unmanned aerial vehicles (UAVs) can be used to resolve this issue and to support the restoring communication infrastructure, as shown in Figure 1. In recent studies, UAVs equipped with a communication relay have been actively studied to temporarily restore the damaged communication infrastructure and provide network services in disaster areas [15,16,17,18,19,20,21,22,23,24,25,26,27]. In [15,16,17,18,19,20], the studies focus on optimizing UAV deployment to enhance communication coverage and efficiency in disaster scenarios. These works address 3D UAV placement, resource allocation, and beamforming techniques for effective communication in diverse disaster areas. In [15], the paper proposes an analytical framework for the optimal deployment of drone base stations in cellular networks. It focuses on balancing coverage and energy efficiency while minimizing the number of UAVs required. The framework adapts to user density variations, making it particularly useful for dynamic disaster environments. The resource allocation and 3D placement of drones is proposed for LTE-based machine-to-machine communications in [16]. It provides algorithms to maximize connectivity and minimize latency, which are crucial for disaster situations. In [17], millimeter-wave base stations are considered, focusing on high-density urban disaster areas. This optimizes the trade-off between throughput and coverage. In [18], the authors investigate the optimal beamforming and deployment of millimeter-wave drone base stations to maximize spectral efficiency. The paper addresses energy efficiency and interference management, providing a strong basis for scalable UAV deployments. A beamforming design for UAVs equipped with reconfigurable intelligent surfaces is introduced in [19]. The proposed algorithm enhances energy efficiency and signal quality. In [20], the paper proposes a swarm-based UAV networking approach using adaptive routing schemes. The proposed approach makes resilience and flexibility, robust coverage and minimal latency in disaster areas. The above papers provide an analytical framework and simulation models including varying disaster environments. However, the above works only validate the framework on the simulations which lack real-world testing and practical challenges.

In [21,22,23,24,25,26,27], the studies focus on integrating UAVs with existing communication networks to restore connectivity rapidly in disaster areas. These works explore multi-UAV cooperation, UAV mesh networking, and interactions with terrestrial networks to bridge communication gaps and improve network resilience in disaster-stricken areas. In [21], the paper proposes a UAV-based approach for post-disaster distribution system restoration, combining communication recovery with power grid restoration. It integrates UAV networks with smart grid systems, making it a unique solution. In [22], the study uses reinforcement learning to optimize UAV network coverage for disaster rescue operations. Its adaptability to dynamic conditions and multi-UAV collaboration is a key strength. In [23], the paper explores UAV-assisted mesh networking for emergency communication, focusing on rapid deployment, scalability, and self-organizing capabilities. In [24], the study proposes multi-UAV cooperation to restore connectivity. It integrates UAVs seamlessly with terrestrial networks, enhancing resilience. In [25], the paper addresses delay-aware UAV computation offloading and communication assistance. It proposes methods to balance communication and computation loads, ensuring efficient resource utilization in disaster scenarios. In [26], the study applies non-orthogonal multiple access (NOMA) techniques to UAV-aided networks, ensuring high-capacity communication in the emergency scenarios. In [27], the paper examines the use of UAVs for post-disaster communication networks, providing comprehensive models for UAV deployment. It is the practical study focusing on real-world applicability; however, it does not address an independent operation of UAV-based communication restoration. The above papers consider a practical solutions for UAV-based communication network restoration. However, the above works are only validated on simulations and lack real-world testing and practical experimental results.

The above existing studies have proposed various technical aspects of UAV-based mobile base stations. However, the previous works did not provide information on whether the proposed UAV-based mobile base station forms an independent mobile network even in disaster scenarios where the connection to the core network is lost. If the core network is disconnected, several critical issues are raised, e.g., the user authentication, resource allocation, and seamless handover, which are caused by the absence of EPC functions in the core network. Hence, in this paper, we propose a UAV-based mobile base station which can operate an independent network even when the core network is disconnected. Furthermore, we provide a prototype and implementation results of UAVs equipped with a mobile base station. We develop a mobile base station based on Public Safety LTE (PS-LTE), which is a wireless communication technology designed for public safety and emergency services. The PS-LTE is the proven standard-based protocol which can provide a secure, reliable, and priority-based communication system for emergency response teams and make it a crucial technology for disaster management. The PS-LTE optimizes the needs of first responders, e.g., police officers, fire departments, and emergency medical teams. Unlike the commercial LTE system, the PS-LTE prioritizes reliability, secure communication, and real-time data transfer during emergencies. The PS-LTE provides the following four key technologies. First, group communication system enabler (GCSE) allows the transmission of group communication content to terminal devices within a specific area. Second, mission critical push to talk (MCPTT) enables one-to-one or group calls with minimal latency by allowing users to communicate by simply pressing a button, similar to a walkie talkie. Third, proximity-based services (ProSe) standardiz communication between nearby devices, supporting direct device-to-device communication for terminals within or outside of the base station’s coverage area. Lastly, isolated E-UTRAN operation for public safety (IOPS) enables a local EPC to provide data access services as a substitute for the core network’s EPC when a wired backhaul issue occurs between the base station and the service core network [28].

In this paper, we develop a UAV-based mobile base station equipped with PS-LTE IOPS functionality. Through this mobile base station, a standalone communication network can be rapidly deployed in the area where existing communication infrastructure has been destroyed, and this restored communication network can rapidly support mission operations. For example, in disaster scenarios where the mobile base station is lost and the interface between the existing base station and the core network is lost, the proposed UAV-based mobile base station can quickly move, i.e., actually fly, to the location of the destroyed original base station. Then, the UAV-based mobile base station replaces the lost mobile base station and establishes an independent mobile network using its built-in EPC functionalities. We develop the prototype of the UAV-based mobile base station and evaluate the prototype in the outdoor environment to investigate whether the prototype works independently without the help of the core network. To the best of our knowledge, this paper is the first to provide an implementation of a UAV-based mobile base station which is equipped with PS-LTE IOPS functionality, enabling an independent communication network to be set up in damaged mobile infrastructure systems.

The rest of this paper is organized as follows: In Section 2, we provide hardware and software design of the proposed UAV-based mobile base station equipped with PS-LTE IOPS functionality. In Section 3, we show a simulation result to achieve the radio coverage of a mobile base station. The prototype of the mobile base station mounted on a UAV can be shown in Section 4. In Section 5, the experimental results are provided, with it being determined that the proposed prototype works very well in an outdoor environment. Finally, we conclude this paper in Section 6.

## 2. System Model

In this section, we provide a hardware and software design of the proposed UAV-based mobile base station equipped with a PS-LTE IOPS functionality. By considering the small size and power consumption of the proposed mobile base station, we expect that the proposed mobile base station can serve a maximum of 100 subscribers. However, it can serve all services that can be applied in PS-LTE services, e.g., push-to-talk, real-time video streaming, coverage extension, etc. The UAV-based mobile base station is equipped with LTE eNB and LTE local EPC, which can make an independent mobile network even when the existing core network is disconnected. The mobile base station is designed to temporarily restore existing infrastructure and to support the rescue teams and disaster recovery efforts in disaster situations. Hence, we assume that only pre-registered devices, e.g., rescue team devices, can be supported by the proposed mobile base station. In the next subsections, we first provide the hardware design and the software design will be followed.

### 2.1. Hardware System

**Figure 2:**
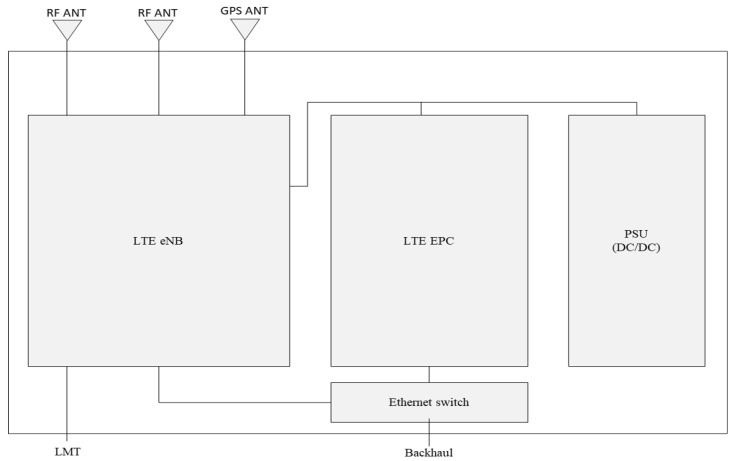
The overall hardware structure of the mobile base station.

The hardware platform of the mobile base station consists of five main components, the LTE eNB, the LTE EPC, the power supply unit (PSU), the GPS antenna, and the Ethernet switch, as depicted in Figure 2. The LTE eNB is the LTE base station that provides the radio interface to the user equipment (UE) to connect to the LTE network. The LTE EPC is a part of the core network of LTE system that provides managing data traffic, signaling, and connectivity between the UE and external networks. The PSU receives +24V DC power from an external power source, and it provides the necessary DC power to each LTE eNB and LTE EPC component. The GPS antenna receives GPS signals that are used to synchronize the internal system clock. The LTE eNB is connected to the LTE EPC via the Ethernet switch.

**Figure 3:**
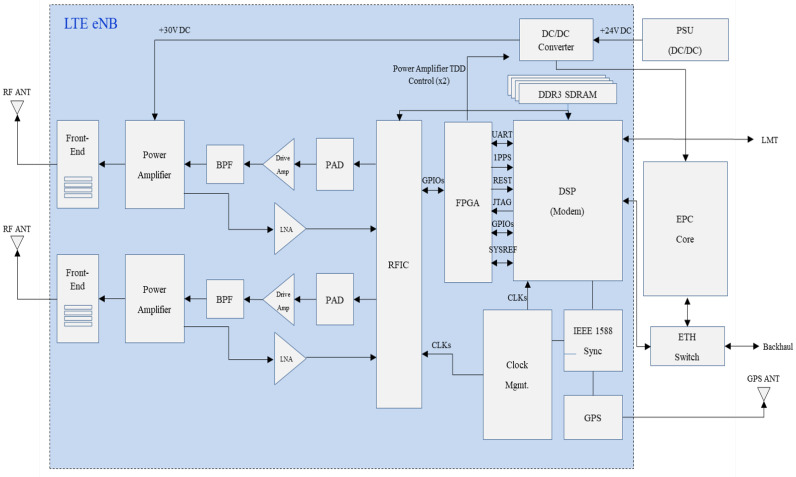
LTE eNB module hardware design.

We show the detail of LTE eNB module in Figure 3. The LTE eNB consists of digital signal processing (DSP), radio frequency integrated-chip (RFIC), power amplifier module (PAM), band-pass filtering (BPF), low-noise amplifier (LNA), and the front-end module. Each component of LTE eNB is a legacy commercial element, while we design and develop a LTE eNB module. The DSP module handles call and network processing as a main processor of LTE eNB component. The RFIC module converts the base-band signal to the high-frequency signal in downlink data flow or it converts the high-frequency signal to the base-band signal in uplink data flow. The PAM amplifies the power of output of RFIC module, i.e., high-frequency converted signal. The BPF selectively passes the RF signal within a specific frequency range. The LNA amplifies weak RF signals with minimal added noise, improving the signal-to-noise ratio before further processing. The front-end module manages the RF signal processing in both the transmission and reception paths.

The uplink and downlink signals are processed through each component in the above LTE eNB module. In uplink signal processing, the RF antenna receives the uplink signal from an external terminal device. The uplink signal passes through the front-end, and this signal is amplified by LNA. Then, this high-frequency signal is converted to the base-band frequency by the RFIC module. The baseband signal is demodulated and decoded in the DSP unit, and these decoded data are delivered to the EPC as an IP packet. In downlink signal processing, the downlink signal follows the reverse path of the uplink processing. First, the downlink IP packet arrives at the EPC. Then, this IP packet is encoded and modulated into an LTE baseband signal in the DSP. The baseband signal is up-converted to a high-frequency RF signal by the RFIC. This high-frequency signal is power amplified and then radiated through each antenna port after passing through the front-end.

**Figure 4:**
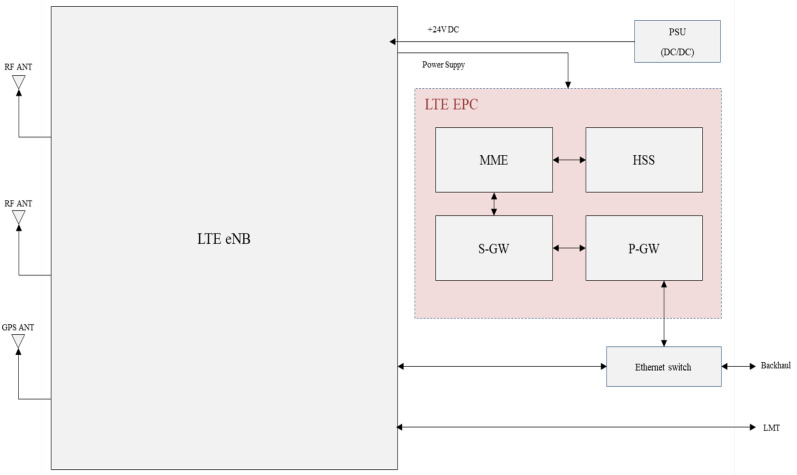
LTE EPC module hardware design.

The LTE EPC consists of four main components, the mobility management entity (MME), home subscriber server (HSS), serving gateway (S-GW), and packet data network gateway (P-GW), as depicted in Figure 4. The MME manages call processing between the mobile base station and terminal devices. It handles non-access stratum (NAS) signaling which is required for terminal devices to connect to the mobile base station, and it controls connections with the packet data network (PDN). The HSS functions as a database that stores key information and subscriber profiles for authentication of each terminal device connecting to the mobile base station. The subscriber profile includes quality of service (QoS) parameters, e.g., priority levels and maximum allowable bandwidth, which are defined by the user’s service agreement. When a terminal device connects to the mobile base station, the HSS provides the necessary authentication keys and subscriber profile information to the MME. The S-GW manages the transport layer of data packets in both uplink and downlink paths, and the P-GW handles the application data processing between the S-GW and the packet data network on the user plane. Also, the P-GW assigns IP addresses to terminal devices.

### 2.2. Software System

**Figure 5:**
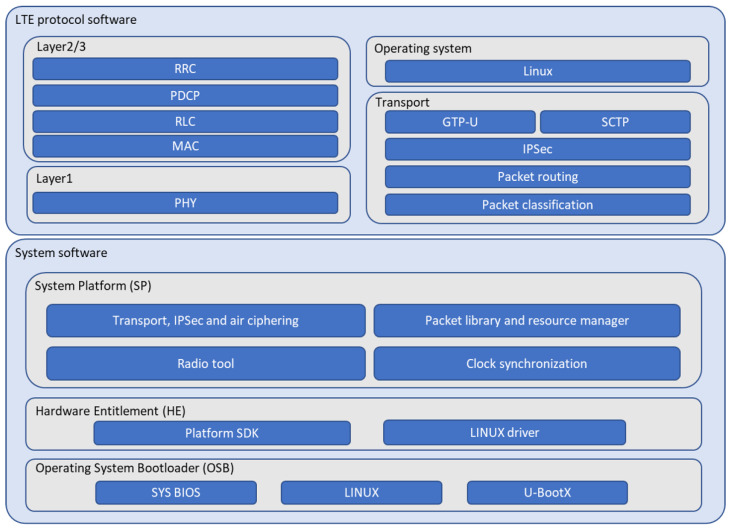
Software module of the mobile base station.

The mobile base station’s software consists of system software and LTE protocol software, and its overall function modules can be depicted as shown in Figure 5. The system software includes the operating system bootloader (OSB), which controls the BIOS and system startup, the hardware entitlement (HE) which enables a software development kit (SDK) and Linux drivers, and the system platform which manages the packet library, the resource management functions, the transport stack for handling packet transmission and reception, and the clock synchronization to ensure time alignment. We develop all the above system software components based on the open source software and standard document, and all the system software components can be summarized as Table 1.

The LTE protocol software is based on 3GPP LTE standards and includes protocol stack, i.e., PHY, MAC, RLC, PDCP, RRC, S1AP, IPSec, SCTP, and GTP-U [29,30,31,32]. The PHY module handles the transmission and reception of LTE downlink (DL) and uplink (UL) frames. The MAC module is responsible for mapping logical channels to physical channels and vice versa. It also supports dynamic or semi-persistent frame scheduling for data frames and implements Hybrid Automatic Repeat Request (HARQ) at the MAC layer. The RLC module manages error detection and retransmission using ARQ and supports PDU concatenation and segmentation. The PDCP module includes functionalities such as packet header compression, ciphering, deciphering, and integrity protection. The RRC module is responsible for connection establishment and release, radio resource management, bearer management, and handover. The S1AP module facilitates inter-operation between the eNB and MME. The transport module includes IPSec for IP packet security processing, SCTP for handling S1 signaling between the eNB and MME as well as X2 signaling, and GTP-U for data packet transmission between the eNB and S-GW. All the above LTE protocol software are summarized in Table 2.

## 3. Simulation Result

In this section, we provide a simulation result to achieve the radio coverage of a mobile base station. We use the Atoll simulator, which is a widely used software tool used for the design, optimization, and simulation of wireless networks, and it supports various network technologies, e.g., LTE, 5G, and IoT networks [33]. The simulation parameters are summarized in Table 3.

We assume that the mobile device is positioned at a height of 1.5 m on the ground, while the base station can be positioned at heights of 100 m, 300 m, and 500 m. The geographic terrain information is mapped to the base station’s location, and the received signal power is analyzed based on the terrain. We use the Okumura–Hata rural model as the wireless channel’s path loss *L*, as shown in Equation (1).

(1) $$\begin{array}{c} L=69.55+26.16\log\left(f\right)-13.82\log\left(h_{t}\right)-h_{r}\left(1.1\log(f)-0.7\right)+1.56\log\left(f\right)-0.8+C, \end{array}$$

where *f* represents the frequency (MHz), $h_{t}$ denotes the height of the transmitting antenna (m), $h_{r}$ is the height of the receiving antenna (m), and *C* is a constant that reflects regional characteristics. The value of *C* varies depending on the type of terrain, such as urban, suburban, or rural areas. The propagation loss value can be calculated by using the Okumura–Hata model with input parameters, i.e., frequency and distance. This model shows that higher propagation loss at higher frequencies, increased loss over greater distances, and varying propagation loss depend on the type of terrain.

**Figure 6:**
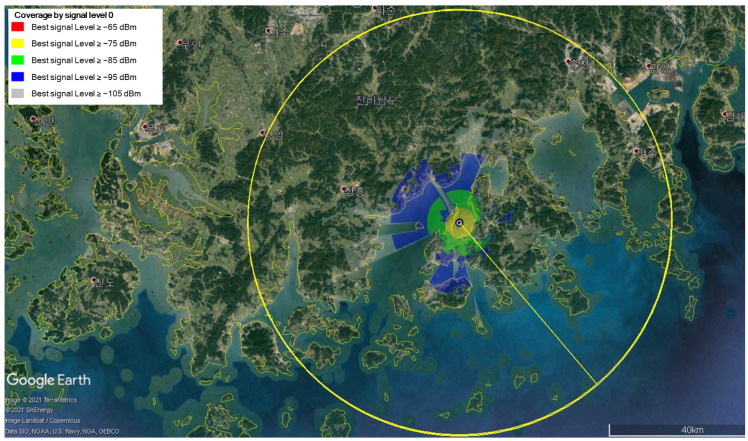
The received signal strength when the altitude of the mobile base station is 100 m.

**Figure 7:**
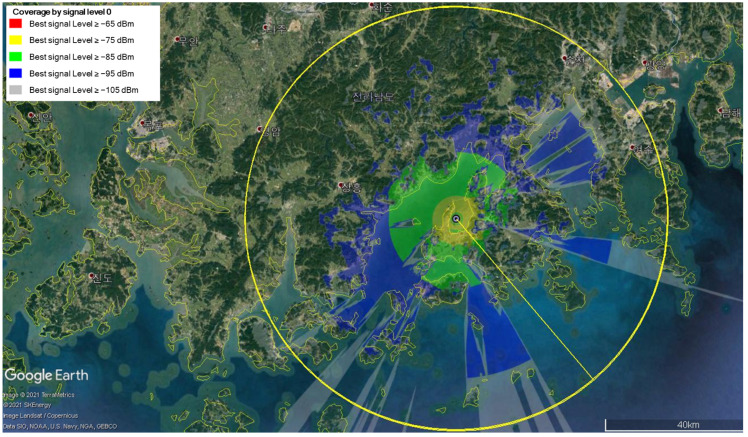
The received signal strength when the altitude of mobile base station is 300 m.

**Figure 8:**
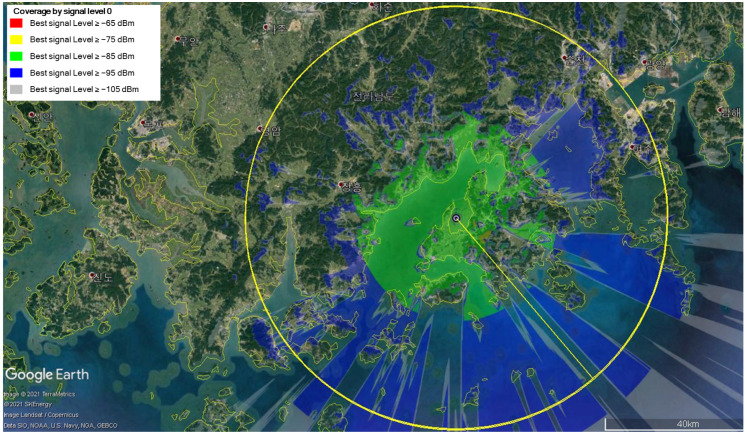
The received signal strength when the altitude of the mobile base station is 500 m.

In Figure 6, Figure 7 and Figure 8, we show the transmission coverage of the proposed mobile base station. Note that the non-English content in the figure is the Korean region name. We can estimate the transmission coverage of the area where the mobile base station experiment would be performed. The mobile base station is equipped with two transmit antennas, each with a maximum transmission power of 36 dBm. We analyze the received signal strength by varying the altitude of the mobile base station at 100 m, 300 m, and 500 m. The received signal strength can be visualized by using the following color scheme: the red color if signals are larger than or equal to 65 dBm, the yellow color if signals are larger than or equal to 75 dBm, the green color if signals are larger than or equal to 85 dBm, the blue color if signals are larger than or equal to 95 dBm, and the gray color if signals are larger than or equal to 105 dBm. Figure 6 shows the signal strength when the mobile base station is at an altitude of 100 m, Figure 7 at 300 m, and Figure 8 at 500 m. The signal strength simulation results show that the received signal strength decreases as the altitude of the mobile base station increases. Since the mobile base station is located in the UAV, the transmission signal has a line of sight (LOS). Hence, the received signal power is more influenced by distance-related attenuation due to the LOS rather than the fading effects caused by the multi-path. This causes the received signal strength to decrease as the altitude of the mobile base station increases.

## 4. Prototype

**Figure 9:**
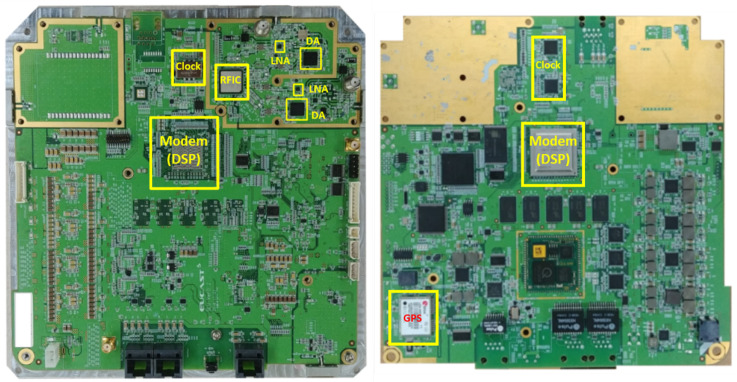
Prototype of eNB module.

**Figure 10:**
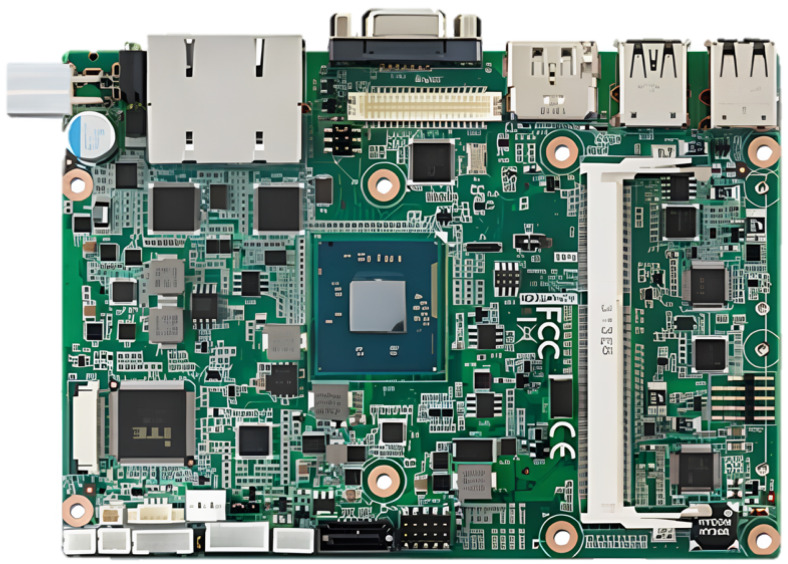
Prototype of EPC module.

**Figure 11:**
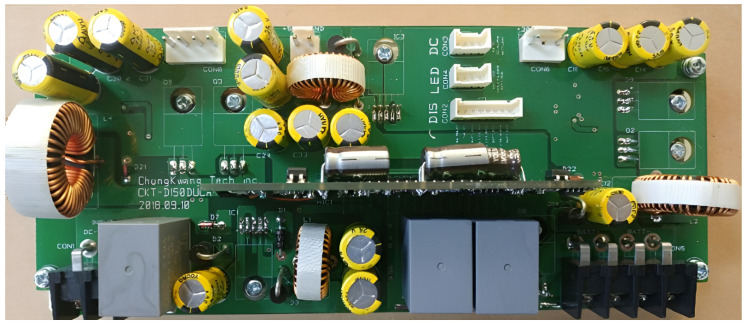
Prototype of DC/DC module.

**Figure 12:**
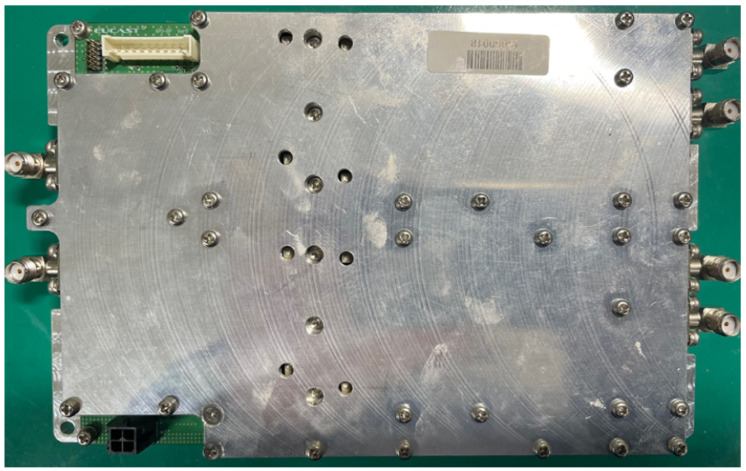
Prototype of power amplifier module.

In this section, we show the prototype of the mobile base station mounted on a UAV, as depicted in Figure 9, Figure 10, Figure 11 and Figure 12. The hardware components of the mobile base station include the eNB module, EPC module, DC/DC power module, and power amplifier module. Figure 9 shows the eNB prototype, which includes DSP, RFIC, LNA, DA, and GPS modules. The DSP module performs modulation and demodulation of LTE baseband signals. The baseband signal can be converted to the RF signal via the RFIC module, and the RFIC signal can be converted to the baseband signal, too. The LNA module amplifies the RF signals received from the BPF and transmits them to the RFIC. Oppositely, the DA module amplifies the RF signals received from the RFIC and transmits them to the BPF module. The clock module generates a time synchronization which is used in all components of the mobile base station, and this clock signal is synchronized with the GPS. The GPS module’s reference synchronization clock is 10 MHz and 1 PPS. Figure 10 shows the EPC prototype. In the EPC module, EPC functions, i.e., MME, HSS, S-GW, and P-GW, are installed on a commercial hardware platform. We use the Intel Atom E3825 board in the prototype. The DC/DC power prototype is depicted in Figure 11. The DC/DC power module receives the external power and converts it to proper voltages available at the mobile base station. In the prototype, the external voltage is +24VDC, and it is converted to +12VDC for the eNB, +12VDC for the EPC, and +30VDC for the power amplifier. Finally, Figure 12 shows the power amplifier prototype, which amplifies the output signal of eNB module and delivers this signal to the antenna.

**Figure 13:**
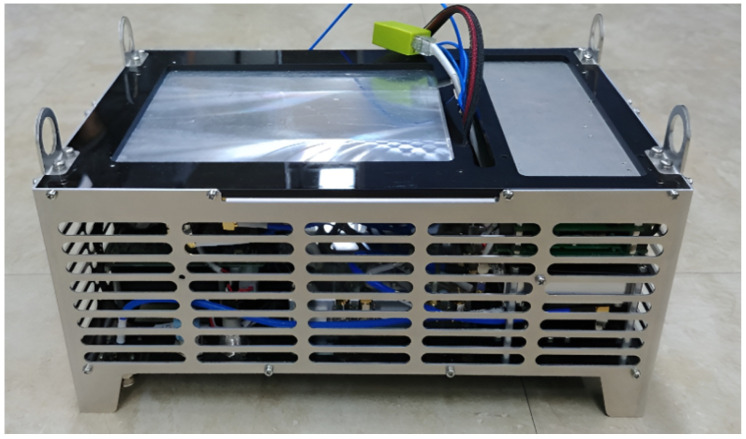
Prototype of mobile base station.

**Figure 14:**
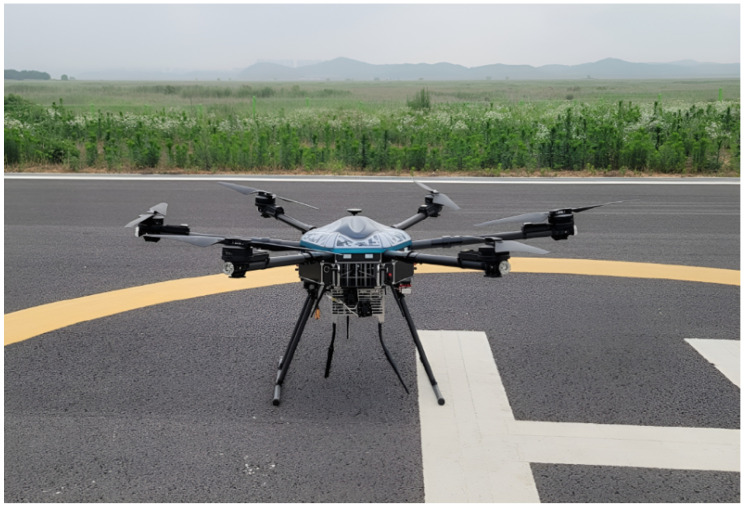
UAV mounted at mobile base station.

The all above mobile base station modules are packaged into the one prototype, as shown in Figure 13, and the UAV with the mounted mobile base station prototype is shown in Figure 14. Since the mobile base station has to be mounted on the UAV, its size and weight should be limited by considering the UAV’s size and payload capacity. The size of the UAV prototype is 181 × 181 × 75 cm^3^, and its payload capacity is 9.3 kg, as summarized in Table 4. The mobile base station is sufficiently small and lightweight to be installed on the UAV considering the UAV’s specification. The size of the mobile base station prototype is 34 × 24 × 15 cm^3^, and its total weight is 7 kg. The input voltage of the prototype is 24 V, and its total power consumption is 130 W. By considering a small size and weight of the mobile base station, it operates continuously around 30 min. The prototype has two transmission antennas, and the antenna transmission power is 4 W for each antenna. We summarize the above specification of the mobile base station in Table 5.

## 5. Experimental Result

**Figure 15:**
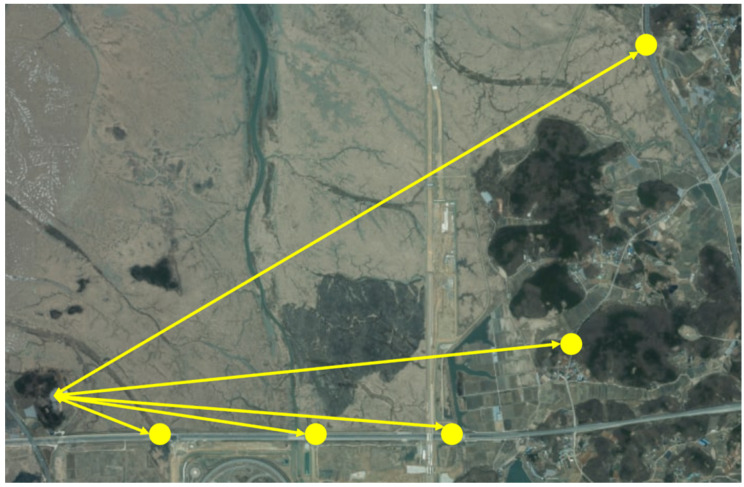
The location of mobile base station.

In this section, we provide the experimental results of the UAV-based mobile base station prototype. The end device is first registered with the mobile base station on the ground. Following the registration process, we verify the communication link between the end device and the mobile base station. Once the link is successfully established, the UAV carrying the mobile base station is elevated to an altitude of 300 m. The mobile base station is positioned at distances ranging from 0 km to 5 km from the end device. Here, a distance of 0 km is defined as the ground location directly beneath the aerial mobile base station. The choice of testing up to 5 km is based on prior simulation results. In the simulation results of the previous section, we evaluate that if the mobile base station is located at an altitude of 300 m, the received signal strength (RSS) remains above −65 dBm within a coverage radius of approximately 5 km. The locations of the mobile base station and the end device are depicted in Figure 15.

To validate whether the mobile base station prototype can operate as an independent LTE communication system, we have a ping test and data transmission evaluations between the one mobile base station and the one end device. Although these experiments represent simple data communication tests, they allowed us to measure the average reference signals’ received power (RSRP) and both downlink (DL) and uplink (UL) data rates. Each data transmission test consists of one-minute trials repeated five times, and the RSRP and data rate are calculated as the average values over a one-minute period.

**Figure 16:**
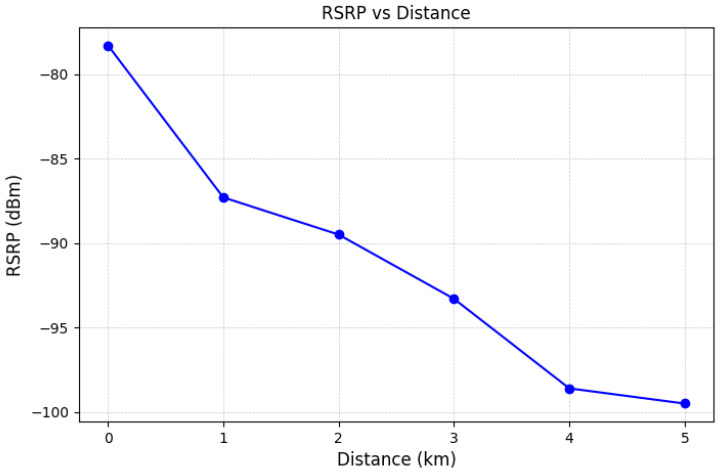
Average RSRP at a distance from the mobile base station.

**Figure 17:**
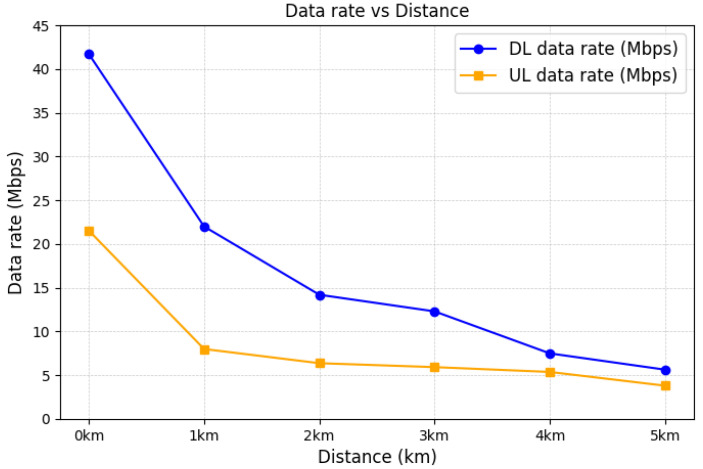
Average data rate at a distance from the mobile base station.

In Figure 16, we show the RSRP at intervals of 1 km, as the distance between the mobile base station and the end device increased from 0 km to 5 km. As the distance between the mobile base station and the end device increases, the RSRP decreases due to the impact of line-of-sight (LOS) conditions and the associated signal attenuation over greater distances. We also show the average DL and UL data rates at intervals of 1 km, as the distance between the mobile base station and the end device increased from 0 km to 5 km, as depicted in Figure 17. The results show a significant reduction in data rates as the distance increases. This is also due to the impact of LOS associated with the signal attenuation over greater distances. At a distance of 1 km, the mobile base station achieves a DL data rate of 22 Mbps and a UL data rate of 8 Mbps. Assuming an average LTE cell radius of approximately 1 km, this result confirms that the mobile base station prototype can sufficiently replicate the functionality of a standard LTE cell. Furthermore, at a distance of 5 km, the mobile base station achieves DL and UL data rates of 5.6 Mbps and 3.8 Mbps, respectively. These data rates are sufficiently high for transmitting critical information in wide-area disaster scenarios. Based on the above experimental results, we show that the proposed mobile base station prototype demonstrates its capability to operate as an independent LTE base station. Moreover, these results confirm that the UAV-based mobile base station prototype can serve as a viable alternative to the existing mobile base stations lost, and it provides sufficient data communication support in wide-area disaster scenarios.

## 6. Conclusions

This paper developed a UAV-based mobile base station to restore communication networks in disaster scenarios. The mobile base station uses PS-LTE technology, which is optimized for emergency services, e.g., police, fire departments, and medical teams. Specifically, we developed a mobile base station including the PS-LTE IOPS functionality to establish an independent communication network even in the case when conventional communication infrastructure is destroyed. We developed the prototype of a UAV-based mobile base station including hardware and software components. Based on the simulation results, we performed outdoor experiments with the mobile base station prototype. The mobile base station prototype successfully operated as a standalone communication unit, with verified data rates of 22 Mbps downlink and 8 Mbps uplink at a 1km distance. Hence, we believe that the proposed mobile base station and its prototype provide an alternative solution to restore communication infrastructure even during periods of disconnection to the conventional communication system.

## Figures and Tables

**Table 1:** System software.

Module	Function
OSB	BIOS, OS Bootloader
HE	SDK, Linux driver
SP	Packet library, resource management, clock synchronization, transport

**Table 2:** LTE protocol software.

Module	Function
PHY	LTE DL, UL frame transmission and reception
MAC	Mapping between logical channel and physical channel, Frame scheduling, HARQ
RLC	ARQ, PDU concatenation, segmentation
PDCP	Packet header compression, ciphering, de-ciphering, integrity protection
RRC	Radio resource management, Radio bearer management, Handover
S1AP	Inter-operation between eNB and MME
IPSec	IP packet security
SCTP	S1/X2 signaling between eNB and MME
GTP-U	Data packet tunneling between eNB and S-GW

**Table 3:** Simulation parameters.

Parameter	DL	UL
Number of transmit antennas	2	1
Bandwidth	10 MHz	10 MHz
Carrier frequency	778 MHz	723 MHz
Maximum transmitter power per antenna	36 dBm	23 dBm
Total transmit power	39 dBm	23 dBm
Transmitter antenna gain	5 dBi	0 dBi
Cable, connector, combiner, body losses	1 dB	0 dB
Transmitter EIRP	43 dBm	23 dBm

**Table 4:** The UAV prototype specification.

Specification	Value
Size	181 × 181 × 75 cm^3^
Weight	33.2 kg
Payload Capacity	9.3 kg

**Table 5:** The mobile base station prototype specification.

Specification	Value
Size	34 × 24 × 15 cm^3^
Weight	7 kg
Input Voltage	24 V
Power Consumption	130 W
Transmission Power	4 W

## Data Availability

Data are contained within the article.
